# Family Caregiver and Clinician Perceptions of Resource Access in Rural Areas

**DOI:** 10.1093/geroni/igaf122.321

**Published:** 2025-12-31

**Authors:** Victoria Ngo, Elizabeth Chamberlin, Elizabeth Marfeo, Steven Shirk, Bret Hicken, Cathy Cruise, Maria Venegas, Lauren Moo

**Affiliations:** VA Bedford Health Care System, Bedford, Massachusetts, United States; VA Bedford Health Care System, Bedford, Massachusetts, United States; Tufts University, Medford, Massachusetts, United States; VA Bedford Health Care System, Bedford, Massachusetts, United States; VHA Office of Rural Health, Salt Lake City, Utah, United States; Veterans Health Administration, Northpoint, New York, United States; Department of Veterans Affairs, Bedford, Massachusetts, United States; VA Bedford Healthcare System, Bedford, Massachusetts, United States

## Abstract

Family caregivers and clinicians in the Veterans Health Administration (VHA/VA) play crucial roles in supporting older Veterans, particularly in rural areas where access to resources can be challenging. A qualitative analysis of interviews with 18 rural family caregivers and 10 clinicians highlights a disconnect between these two groups regarding resource access. Thematic analysis revealed that clinicians assume that patients are being provided services through social workers, believing that these professionals effectively connect families to needed services. However, when asked, rural family caregivers report being unaware of many available VA programs and services (e.g., VA Caregiver Support Program, mileage reimbursement), leaving them feeling unsupported and unsure of where to seek help. This gap in knowledge contributes to frustration and missed opportunities for essential support services. The differing perceptions between clinicians and family caregivers highlight systemic barriers in resource navigation for older veterans. While clinicians rely on social workers as a point of contact for connecting families with community services, family caregivers in rural areas may struggle with limited awareness, inconsistent outreach, or difficulty in accessing social support altogether. This disconnect underscores the need for improved communication strategies within the VA system to ensure family caregivers are informed and empowered to access resources. Efforts such as proactive outreach, improved caregiver and clinician education, and clearer roles within care teams could bridge this gap and improve outcomes for both caregivers and the older veterans they support.

